# Photodynamic therapy guidelines for the management of oral leucoplakia

**DOI:** 10.1038/s41368-019-0047-0

**Published:** 2019-04-11

**Authors:** Qianming Chen, Hongxia Dan, Fan Tang, Jiongke Wang, Xiaoying Li, Junxin Cheng, Hang Zhao, Xin Zeng

**Affiliations:** State Key Laboratory of Oral Diseases, National Clinical Research Center for Oral Diseases, Chinese Academy of Medical Sciences Research Unit of Oral Carcinogenesis and Management, West China Hospital of Stomatology, Sichuan University, 610041 Chengdu, Sichuan China

**Keywords:** Mucositis, Combination drug therapy

## Abstract

With recent developments in photosensitizers and light delivery systems, topical 5-aminolevulinic acid-mediated photodynamic therapy (ALA-PDT) has become the fourth alternative therapeutic approach in the management of oral leucoplakia (OLK) due to its minimally invasive nature, efficacy, and low risk of systemic side effects and disfigurement. This report presents step-by-step guidelines for applying topical ALA-PDT in the management of OLK based on both the clinical experience of the authors and a systematic review of the current literature. Studies using protocols with standardized parameters and randomized clinical trials at multiple centres with adequate sample sizes and both interim and long-term follow-ups are needed before universally applicable guidelines can be produced in this field.

## Oral lesions: a lighter touch for treatment

Light-mediated therapy is a promising approach for treatment of oral leukoplakia (OLK), white patches inside the mouth that cannot be wiped away and that can become cancerous. Surgical removal carries a risk of damaging delicate facial structures, or causing potentially disfiguring scarring. Hang Zhao and Xin Zeng at the West China Hospital of Stomatology and coworkers have reviewed use of photodynamic therapy (PDT), a minimally invasive alternative to treat OLK. For treatment, the lesion is coated with a photosensitizer solution, then illuminated with a laser (or LED), triggering release of reactive oxygen species, which kill nearby cells. The treatment can be focused very precisely on a specific tissue, without damaging other cells. The researchers recommend further research to determine long-term effectiveness against OLK, and to develop standardized protocols for wider application of this promising method.

## Oral leucoplakia and management approaches

Oral leucoplakia (OLK) is most likely to be defined as a predominantly white patch or plaque or lesion of the oral mucosa that cannot be wiped away and is not clinically or histologically characterized as any other definable disorder,^1–3^ such as oral candidiasis, lupus erythaematosus, lichen planus, hairy leucoplakia, frictional keratosis, nicotinic stomatitis, or leukoedema. Since OLK carries an increased risk of cancer development either in or close to the area of the lesion or elsewhere in the oral cavity or the head-and-neck region, it is listed among potentially malignant oral lesions. The definition of OLK is based on the integration of the definitions of the Society of Oral Medicine, Chinese Stomatological Association and World Health Organization (WHO),^1–4^ although some discussions regarding the definitions are still ongoing.^5–8^ A two-stage system, i.e., one consisting of provisional and definitive definitions, is recommended for the diagnosis of OLK depending on the circumstances of the examination of the oral mucosa and the availability of diagnostic examinations.^5,7^ A provisional diagnosis of OLK is made when a lesion found on clinical examination cannot be clearly diagnosed as any other disease of the oral mucosa and has a white appearance at the first visit, while a definitive diagnosis of OLK is made based on retrospective observations after successful elimination of the possible causative factors within a period of 4–8 weeks, together with the results of a histopathological examination.^1,5,7^ The aetiology of leucoplakia remains unclear, although risk factors, such as tobacco and alcohol use, oral candidiasis, human papilloma virus (HPV) infection, microcirculation disturbances and fat-soluble vitamin deficiency, have been suggested.^1,5,7^

OLK has gained attention because of its potential risk for malignant transformation,^9^ which occurs at an estimated rate of approximately 0.13%–34%, based on a systematic review of 24 retrospective studies.^10^ In a hospital-based investigation of a Chinese population, the malignant transformation rate of OLK ranged from 4 to 13%.^11^ Traditionally, OLK is clinically divided into two types, homogeneous and nonhomogeneous, and further into different subtypes;^3,5^ nonhomogeneous OLK has a higher risk of malignant transformation.^3,5,10^ Therefore, although the main purpose of the clinical management of OLK is to prevent malignant transformation,^12^ the reduction or elimination of the lesion is also desired because of the biological and clinical features mentioned above.^9^

Traditionally, there are two categories of treatment approaches for OLK, non-surgical and surgical; however, there is insufficient evidence to determine which approach is better because the outcomes seem to vary among studies and it is difficult to find well-designed studies with long-term follow-up periods.^9,12^ Chemoprevention or observation without intervention fall into the former category. However, randomized controlled trials have demonstrated no promising evidence regarding the prevention of malignant transformation and recurrence of OLK by chemoprevention.^13^ Surgical methods include traditional excision, electrocauterization, carbon dioxide (CO_2_) laser ablation and cryosurgery. However, invasive methods are less feasible when the lesions are extensive or located at certain anatomical sites with underlying functional structures.^9,12^ Postoperative pain, oedema, and considerable scarring also prevent such methods from being preferred by patients.^9,12^ Therefore, there is a need for alternative treatment approaches for OLK with better efficacy and fewer adverse effects in terms of both bodily functions and disfigurement.^3^ Photodynamic therapy (PDT) has promise as one such alternative treatment.

## Concepts of PDT

### PDT

Photodynamic therapy (PDT) is an alternative minimally invasive approach that has gained attention worldwide in the last two decades. It has been widely used to treat precancerous lesions and cancers of the skin, digestive tract, and genitourinary mucosa, as well as tumours and vascular malformations in other organs. In the oral cavity, PDT has been used for the management of oral cancer and potentially malignant disorders, especially OLK. It has shown high selectivity and repeatability for the treatment of OLK.^14^ PDT has a low morbidity rate and results in good functional and cosmetic outcomes with minimal scarring, which makes it valuable for treating OLK in regions with underlying functional structures.^14,15^ PDT is expected to become a routine or auxiliary treatment for OLK.

**Fig. 1:**
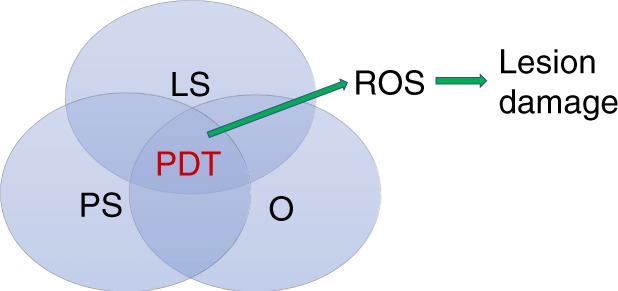
The three fundamental elements of PDT. PDT, photodynamic therapy; LS, light source; PS, photosensitizer; O, oxygen

**Fig. 2:**
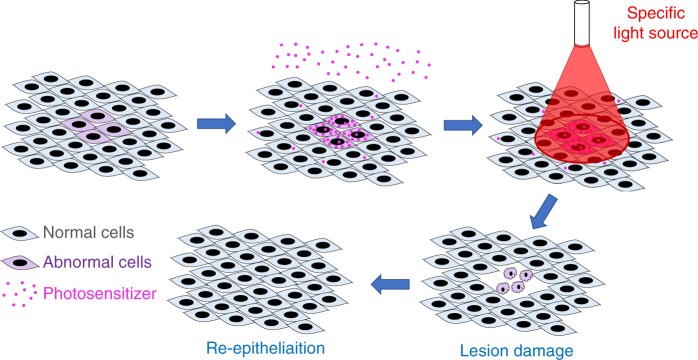
Schematic diagram of photodynamic therapy (PDT). When a photosensitizer is applied to the target lesion and surrounding tissue, it selectively accumulates in cells with abnormal proliferation, and irradiation of the target lesion with light of a specific wavelength will then lead to light-induced damage to these cells without much influence on normal cells. Finally, wound healing occurs at the treated site, and the damaged abnormal cells are replaced by normal cells

The three fundamental elements of PDT are oxygen, a photosensitizer and a specific wavelength of visible light^15^ (Fig. 1). Specifically, the photosensitizer is activated by the light and causes a series of photochemical and photobiological reactions, resulting in irreversible damage to and eventually the death of abnormally proliferative cells^15^ (Fig. 2). Since PDT is a cold photochemical process, there is no tissue heating, and connective tissues, such as collagen and elastin, at the treated site are mainly unaffected; therefore, there is much less risk of damaging the integrity of underlying functional structures than there is with thermal laser techniques and other invasive approaches.^15^

### Oxygen

Without oxygen, PDT would not produce cytotoxic effects.^16^ When a photosensitizer molecule within the cell of a lesion absorbs a photon, the light excites the photosensitizer molecule from its stable ground state to a short-lived unstable singlet state and a relatively long-lived triplet state.^16^ To restore the stable ground state, the activated molecule transfers energy to oxygen, resulting in the formation of reactive oxygen species (ROS).^15,17^ ROS are highly cytotoxic, have a short lifetime (<0.04 µs) and a short radius of action (<0.02 µm), and they contribute to destruction of the lesion, which manifests as swelling and tissue necrosis.^15^ This tissue is finally resorbed, and normal healing and re-epithelialization occurs at the treated site.

### Photosensitizer

5-Aminolevulinic acid (5-ALA), a second-generation photosensitizer, has a low molecular weight, a short period of phototoxicity (24–48 h), good tissue penetration, and a high singlet oxygen yield. It is the most widely used photosensitizer for the treatment of OLK by PDT, although the use of other same-generation photosensitizers, such as hypericin, phthalocyanine, benzoporphyrin derivatives and meta-tetra(hydroxyphenyl)chlorin derivatives, has also been reported in the literature.^18^

ALA is water soluble and can be administered intravenously, orally or locally. ALA cannot produce ROS by itself because it is only a precursor of porphyrin. Exogenous ALA enters cells and is converted endogenously into porphyrin IX (PpIX) via the porphyrin–haem pathway. Once PpIX is exposed to visible light including its absorption peaks at 400–410 nm and 635 nm, ROS are generated. ALA demonstrates excellent tissue selectivity and is preferred by proliferative epithelial cells, probably because the defective epidermal barrier protects the exogenous ALA from being absorbed and slows the conversion of PpIX into haem.^19^

### Light sources

It is preferable to use a laser, i.e., coherent light, as the source of irradiation in PDT for OLK, especially with recent advances in laser devices, such as semiconductor lasers (e.g., diode lasers with wavelengths from 600 to 950 nm), argon lasers (448 to 514.5 nm), and solid-state lasers, such as Nd/YAG lasers (1 064, 532, 355 or 266 nm),^15,16^ because monochromatic light is delivered via an optical fibre, which provides a more stable beam and allows the easy calculation of light dosimetry and irradiation with the optimal wavelength to a specific photosensitizer. However, these kinds of devices are expensive. Alternatively, high-power light-emitting diodes (LEDs), which provide another kind of coherent light, can also be used as a source for PDT.^16^ The wavelength of light produced by LEDs ranges from 350 to 1100 nm, and LED devices are portable and relatively inexpensive. Furthermore, the use of non-coherent light sources, such as conventional lamps, was reported in the literature as the earliest light sources for PDT.^16^

Principally, the most appropriate wavelength for PDT is 600–800 nm, which is known as the “therapeutic window”, because shorter wavelengths have shallower tissue penetration depths, while longer wavelengths may have a lower yield of ROS.^14,16^ With regard to the selection of light devices, several aspects should be taken into consideration, including features of the lesion (tissue, size, location and accessibility), photosensitizer (absorption spectrum and mode of administration) and the light source (cost and availability).^14,16^ However, a systematic review of the literature has shown that there are no significant differences in the efficacy of PDT for OLK using laser sources and LEDs.^14^

It is important to consider the dose or fluence of light.^19^ The following formula represents the relationship between fluence (*ρ*, J•cm^−2^), irradiance power (*P*, mW•cm^−2^), irradiance diameter (cm) and time (*t*, *s*): *ρ* = 4*Pt*/*πd*^2^. The most common dose of light is 100 J•cm^−2^, which is sufficiently powerful for a 635-nm light source to produce ROS; although various light doses are used, those in PDT for OLK range from 50 to 200 J•cm^−2^.^14^

## Clinical procedures

### Preoperative care

**Fig. 3:**
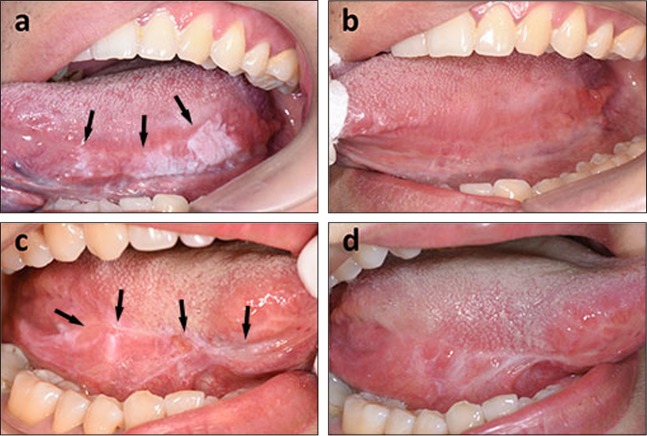
Clinical effects of photodynamic therapy (PDT) on oral leucoplakia (OLK). A patient with a primary OLK lesion (**a**) and the appearance of the site one month after PDT (**b**). No recurrence at the 6-month follow-up; the site of recurrent OLK after scalpel excision (**c**) and its appearance 1 month after PDT, with some scarring from the surgery before PDT (**d**). No recurrence at the 8-month follow-up. The lesions are indicated by black arrowheads

Any patients with OLK (Fig. 3a), including recurrence after scalpel excision (Fig. 3c), laser therapy and cryotherapy, can be treated by PDT. PDT has been recommended as the first-line treatment of choice for some special types of OLK, such as erythroleucoplakia and oral verrucous hyperplasia.^20,21^ However, it is imperative to obtain a thorough patient medical history. A history of porphyria, coagulopathy, pregnancy, uncontrolled severe systemic disorders (such as uncontrolled hypertension, heart disease, diabetes, severe liver and kidney damage, or malignant tumours) and any history of allergy to light, porphyrin or anaesthesia agents are contraindications. Target lesions should be biopsied to determine the degree of dysplasia and whether there is inflammation. In addition, the clinical and histopathological characteristics of the patients, including sex, age, lesion size and type, and grade of dysplasia, should be carefully recorded. Images of the lesions should be captured and stored, as appropriate.

The doctor should introduce the optional treatment schemes, with the advantages and disadvantages, to patients on the basis of strictly mastering the operating techniques, indications and contraindications of PDT so that patients can fully understand the treatment objective, treatment plan, possible results, possible adverse reactions and countermeasures.

The nursing staff is responsible for preparing the materials needed for PDT, carefully double checking the patient’s name, sex, case file, diagnosis and target lesion, among other parameters, and assisting in the procedure.

The patient should provide written consent for the surgical treatment, indicating their full understanding of the treatment purpose, treatment plan, possible results, possible adverse reactions and countermeasures. In addition, the whole blood cell counts, glucose level, blood coagulation time and liver and kidney function, among other blood indices, should be determined before the procedure. Treatment should be avoided when patients have an empty stomach or are exhausted.

PDT needs to be carried out in a dark room; therefore, a strict light-proof environment should be established in the treatment room. To this end, it is required that the luminance of the treatment room is stable under different external environments; curtains consisting of double-layered blackout cloth with a strong light-shielding ability will minimize the interference of external light.

Materials related to treatment should be double checked before the procedure, such as the photosensitizer, e.g., ALA, the settings of the PDT device (e.g., wavelength, 630 nm± 5 nm; output power, 0.1 –2 W; adjustable semiconductors) and optical fibres (e.g., microlens optical fibres), as well as general materials, such as oral disposable examination trays, disposable mouthwash cups, disposable syringes, local anaesthetics, sterile cotton rolls and balls, disposable saliva aspirators, disinfection appliances, goggles, sterile isolation films, 0.1% chlorhexidine gargling solution, and medical swabs.

### Operative care

After entering the treatment room, the patient should rest in a quiet environment for 5–10 min and take a sitting position for blood pressure and heart rate measurements. We recommend treating patients with systolic pressure ≤140 mmHg, diastolic pressure ≤90 mmHg and heart rate ≤100 beats per min.

The nursing staff should record the target area in detail and keep it on file. The nursing staff should also guide the patient through an oral cleaning via gargling with 0.1% chlorhexidine solution for 1 min.

To prepare the photosensitizer solution, the nursing staff should dissolve ALA with sterile water to yield a 20% aqueous solution immediately before use.

Then, a thin cotton swab soaked with the prepared photosensitizer solution is gently placed over the lesion after salivary isolation is established. In addition, a starch film is layered on top of the cotton swab to improve adhesion of the photosensitizer to the oral mucosa. Finally, the target area is covered with food-grade cling film and thick gauze to protect the photosensitizer from dilution by saliva. The scope of the wet cotton swab should exceed the edge of the intended treatment site by 3–5 mm. To increase the penetration of ALA into the tissue, some tools, such as plum-blossom needles, can be utilized for assistance before the incubation.^22^ ALA can be administered by intralesional injection when the lesion is likely to be affected by the secretion of saliva or movement of the tongue.^23^

After incubation for 2–3 h, the swab is removed, and the illumination reaction is tested by UV light (wavelength, 370–470 nm). Then, the patient gargles with water to remove the unabsorbed photosensitizer and cleans the lesion surface again before local anaesthesia using 2% lidocaine or 4% primacaine is performed. We recommend that the patient’s name, sex, age and target sites be double checked once more before the anaesthesia.

Regarding the treatment parameters, based on our own experience, together with reports in the literature,^17^ laser irradiation is performed using a semiconductor laser light source at 630 nm ± 5 nm. A power of 100 mW•cm^−2^ is recommended; each 3-min irradiation session is followed by 3 min of rest to maintain effective intracellular oxygen concentrations until the total light exposure dose reaches 100 J•cm^−2^.

Eye protection is very important during the procedure. After setting the parameters of the device, the operator, assistant and patient should wear protective goggles. In addition, the patient should keep both eyes closed during the procedure to avoid unnecessary stimulation.

During laser exposure, to achieve even irradiation of the target lesion, the laser beam should be as perpendicular to the surface of the lesion as possible. The distance between the end of the optical fibre and the surface of the lesion needs to be appropriate to ensure efficacy. Moreover, the parameters and the patient’s reactions should be observed, recorded in detail and handled in a timely manner if necessary.

The lesion should be treated once every 2–3 weeks, depending on the healing of the lesion.

### Postoperation

The patients should be instructed to keep their mouth clean and to avoid irritating foods and drinks. Moreover, the PDT-treated area should not be exposed to light within 48 h after the treatment. If the lesion is located in an exposed site (such as the lips), this protection must be prolonged to last the entire treatment.

Patients should be asked to report any adverse reactions to the medical personnel, and a visual analogue scale (VAS) should be used to evaluate post-treatment pain.

Topical 0.01% dexamethasone paste and 0.1% chlorhexidine gargling solution are usually prescribed to reduce inflammation after the treatment.

The treatment response is usually recorded 4 weeks after the last treatment (Figs. 3b, d). The lesion response is designated a complete response (CR) if the visible lesion disappears, a partial response (PR) if a reduction in size of at least 20% occurs and no response (NR) if a reduction in size of <20% or an increase in size occurs. The total response (TR) rate was calculated using the following formula: (CR+PR)/(CR+NR+PR)×100%. The reported response rate of OLK to ALA-PDT varies from 50% to 100%, and the CR rate ranges from 16.49% to 88.89%.^14,24^ The recurrence rate ranges from 0% to 41% over a follow-up period of 1–30 months.^14,23,25–28^

### Adverse reactions and management

The most common adverse reactions to PDT for OLK are mild to moderate pain, hyperaemia, oedema, erosion, ulceration and bleeding at the treated area and in the surrounding tissues. Mild cases need no further treatment and can heal spontaneously, while in severe cases, symptoms can be reduced by prescribing anti-inflammatory and antiseptic drugs, such as 0.1% chlorhexidine gargling solution, and glucocorticoid preparations, such as prednisolone solution for topical application. In cases of severe pain, compound benzocaine gel can also be applied topically. In cases of more extensive erosion and ulceration, short-term (3–5 days) and low-dose (15–30 mg) oral prednisone acetate tablets can be considered.

Light sensitivity is another very common side effect of the treatment, which manifests as maculae, papules, blisters, and erosion, among others, appearing on the PDT-treated area after exposure to strong sunlight or indoor light. To prevent such reactions, strict avoidance measures should be taken. The patient should prevent direct exposure of the PDT-treated area to strong sunlight or indoor light and wear protective equipment if necessary, such as a parasol or mask. If the treated area is located at an exposed sites, such as the lip, the lesion should be strictly prevented from being exposed to light for 48 h after the treatment. Furthermore, this exposure prevention should be continued as much as possible until the end of the entire treatment to reduce the generation of pigmentation. If the patient fails to prevent sun exposure as required and the above symptoms occur, then treatment includes getting out of the lighted area and seeking medical attention immediately, taking oral antihistamines, such as cetirizine, gargling with anti-inflammatory anticorrosive drugs, such as 0.1% chlorhexidine, dressing the lesion topically with glucocorticoid preparations, such as prednisolone solution, and visiting a dermatologist immediately if skin damage occurs.

## Future directions

With recent developments in photosensitizers and light delivery systems, topical ALA-PDT, a minimally invasive technique, has become the fourth alternative therapeutic modality, following chemotherapy, surgery, and CO_2_ laser ablation or cryosurgery, in the management of OLK in terms of its efficacy as well as its low risk of systemic side effects and disfigurement. This technique can be used  to treat large and recurrent lesions and has minimal impact on patients in the short and long term. It is simple, can be carried out in most outpatient clinics and is highly accepted by patients.

However, limited data are available on the efficacy of PDT in the management of OLK. Studies with standardized parameters and protocols and randomized clinical trials at multiple centres with adequate sample sizes and interim and long-term follow-up periods are needed before more general guidelines can be produced for the management of OLK. Specifically, further studies should investigate the effects of ALA-PDT on malignant transformation, methods to improve the efficacy of ALA-PDT in the treatment of OLK and the efficacy of PDT compared with various other therapies.
